# Genome Sequences of *Arthrobacter globiformis* Phages Donatella, JellyBread, and LadyJasley

**DOI:** 10.17912/micropub.biology.001878

**Published:** 2025-11-05

**Authors:** Eric Sakowski, Katherine Alfaro, Aliyah Ambriz, Savannah Brake, Sara Brennan, Korri Brown, Arabella Canby, Jessica Castillo, Vincent Desautels, Thalia Flores, Samantha Hackley, Maurece Jean-Brian, Isaac Jimenez, McKayla Lee, Lourdes Lozano-Rojas, Anita Manglona, Shaniah Mayers, Payton Murchison Whiteside, Marissa Nittinger, Eddy Nkwepo, Reese Olenik, Andrew Ramirez, Srishti Sanjeevkumar, Kaylah Scott, Abigail Sies, Hailey Sprow-McCoy, Anika Sproxton, Estefania Tapia, Jasmin Trentler, Jalia Watson, Michaela West, Geraldine Vilmen

**Affiliations:** 1 Department of Science, Mount St. Mary's University, Emmitsburg, Maryland, United States

## Abstract

Bacteriophages Donatella, JellyBread, and LadyJasley are novel phages infecting *Arthrobacter globiformis* B-2979. Donatella and JellyBread have siphoviral morphotypes and were assigned to clusters FF and AY, respectively, with genome lengths of 42,875bp and 53,425bp. LadyJasley has a podoviral morphotype, a genome length of 45,576bp, and was assigned to cluster AV.

**Figure 1. Transmission electron microscopy images of A) phage Donatella; B) phage JellyBread; and C) phage LadyJasley:**
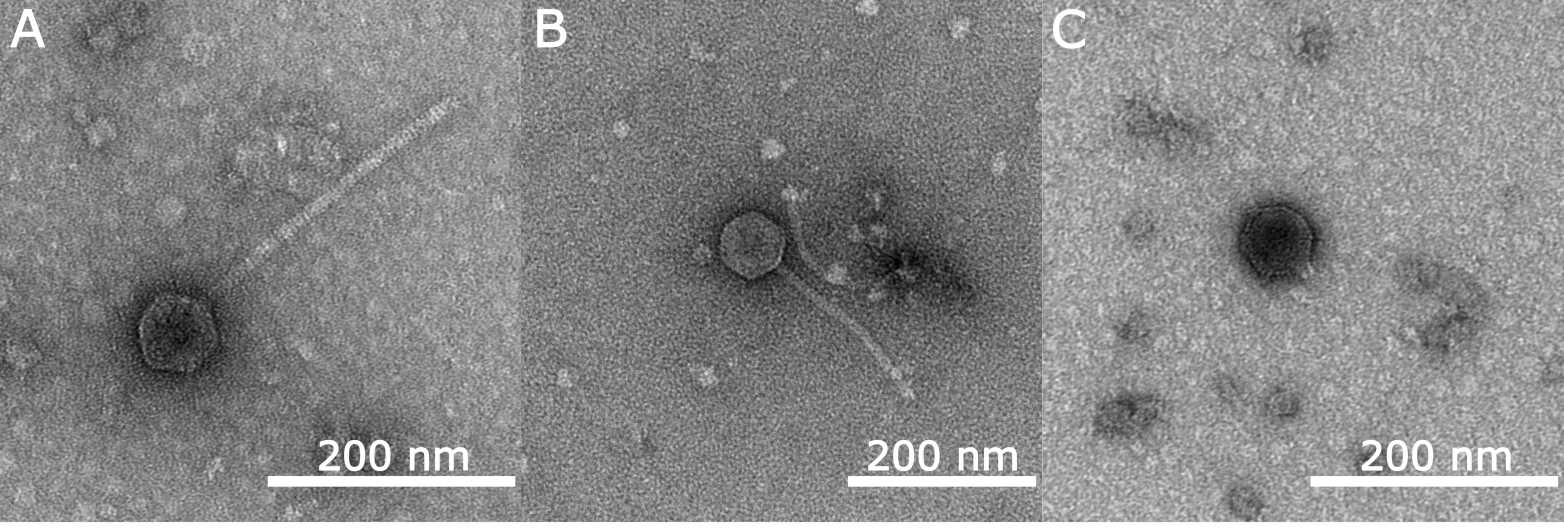
Phages Donatella (A) and JellyBread (B) have a siphoviral morphology with a polyhedral capsid and a long, flexible tail. Phage LadyJasley (C) has a podoviral morphology with a polyhedral capsid and a short, non-contractile tail. Scale bar is 200 nm.

## Description

Viruses are the most abundant biological entities on Earth and a source of novel genetic diversity (Dion et al., 2020). Bacteriophages – viruses that infect bacteria – have numerous applications in biotechnology and medicine, including their use as potential therapeutics for treating bacterial infections (i.e. phage therapy) (Abril et al., 2022). In this study, we isolated and characterized three novel bacteriophages that infect *Arthrobacter globiformis* B-2979.

Bacteriophages Donatella, JellyBread, and LadyJasley were isolated from soil samples collected at Mount St. Mary's University in Emmitsburg, MD (39.68N, 77.35W). Phage JellyBread was isolated in Fall 2023, while phages Donatella and LadyJasley were isolated in Fall 2024. All three phages were isolated on bacterial host *Arthrobacter globiformis* B-2979 using an enrichment protocol. Briefly, soil samples were combined with peptone-yeast calcium (PYCa) liquid media and shaken for one hour at 250 rpm. Samples were spun for five minutes at 3,220 xg, and the supernatant was filtered through a 0.22 µm pore size PES syringe filter. Following filtration, samples were inoculated with *A. globiformis* B-2979 and incubated with shaking for 48 hours at 30°C. The enriched samples were refiltered, plated in top agar containing *A. globiformis* B-2979, and incubated at 30°C. Plaques for phages Donatella, JellyBread, and LadyJasley were purified by three successive rounds of plaque assays (Zorawik et al., 2024). Following 24 hours of incubation at 30˚C, phage Donatella produces turbid plaques 0.5-1mm in diameter (n = 4); phage JellyBread produces turbid plaques 0.5-1.5mm in diameter (n = 3); and phage LadyJasley produces clear plaques 1-2mm in diameter (n = 3). High titer lysates were prepared for each phage and used for transmission electron microscopy (TEM) and DNA extraction for genomic characterization. TEM images identified phages Donatella and JellyBread as possessing siphoviral morphologies, with capsid diameters and tail lengths of 70 ± 4 nm and 257 ± 5 nm (n = 5) for Donatella; and 76 ± 2 nm and 205 ± 8 nm for JellyBread (n = 3), respectively. Phage LadyJasley has a podoviral morphology with a capsid diameter of 61 ± 4 nm and a tail length of 12 ± 1 nm (n = 3) (Fig. 1).

Phage DNA was isolated from high titer lysates by degradation of exogenous (host) DNA via DNase and RNase treatment, followed by DNA extraction and purification using the Zymo Quick-DNA Viral Kit following the manufacturer's instructions. The phage DNA library was prepared using the NEB Ultra II Library Kit and sequenced with an Illumina NextSeq 1000 using 100bp single-end reads. Raw reads were trimmed and filtered with cutadapt 4.7 (Martin, 2011) using the option: –nextseq-trim 30; and skewer 0.2.2 (Jiang et al., 2014) using the options: -q 20 -Q 30 -n -l 50. Trimmed and filtered reads were assembled with Newbler v2.9 (Margulies et al., 2005) using default parameters. Assembled genomes were checked for completeness with Consed v29 (Gordon et al., 1998). Sequencing data and genome characteristics are presented in Table 1. Of note is the GC content for LadyJasley (44.8%), which is the second lowest of all 630 sequenced *Arthrobacter* phages in the phagesDB Actinobacteriophage database ( https://phagesDB.org ) (Russell and Hatfull, 2017) to date; this GC content is also significantly lower than that of *A. globiformis* (65.9%) (Sahoo et al., 2019), suggesting that the isolation host may not be its natural host.

All three phage genomes were annotated in DNA Master v5.23.2 (Pope and Jacobs-Sera, 2017). Open reading frames (ORFs) were predicted using Glimmer v3.02 (Delcher et al., 1999) and GeneMark v2.5 (Lukashin and Borodovsky, 1998). Putative functions were assigned using BLASTP (Altschul et al., 1990) by searching translated ORF sequences against the NCBI NR and Actinobacteriophage (Russell and Hatfull, 2017) databases. Additionally, translated ORFs were queried using NCBI Conserved Domain BLAST v3.21 (Marchler-Bauer et al., 2007); and HHpred (Söding et al., 2005) against the PDB_mmCIF70 and Pfam-A_v37 databases. tRNAs were identified by Aragorn v1.2.41 (Laslett and Canback, 2004) and tRNAscan-SE v2.0 (Chan et al., 2021). Putative membrane proteins were predicted with TMHMM v1.0.42 (Hallgren et al., 2022) using default parameters. Phages Donatella, JellyBread, and LadyJasley were assigned to clusters FF, AY, and AV, respectively, based on gene content similarity (≥ 35%) to phages in the phagesDB Actinobacteriophage database ( https://phagesDB.org ) (Pope et al., 2017; Russell and Hatfull, 2017). Phage Donatella contains 68 putative protein coding genes and one tRNA. Of the 68 putative protein coding genes, 40 could be assigned a function. These include two tyrosine integrase genes, suggesting that Donatella is a temperate phage; this is consistent with the isolation of lysogens for another cluster FF phage (Wise and Sinavathan, 2025). Phage JellyBread encodes 93 putative protein coding genes, including 42 that could be assigned a function, and two tRNAs. Like Donatella, phage JellyBread contains two tyrosine integrase genes and is also predicted to be a temperate phage. It also contains several genes involved in DNA metabolism and regulation, such as a NrdH-like glutaredoxin and a DNA methyltransferase. Phage LadyJasley has 60 putative protein coding genes, only 20 of which could be assigned a function. No tRNAs or integrase genes were identified (Table 1).

Putative protein coding genes in each phage genome were assigned to phage gene families (phams) according to amino acid similarity (Cresawn et al., 2011). For phage Donatella, only one pham is not present in any other cluster FF phage members to date, while phage JellyBread contains eight phams that are absent in all other current cluster AY phages (Table 1). In contrast, over half of LadyJasley's phams (34) are not observed in any other cluster AV members to date (Table 1), including 29 phams with no other phage representatives in the Actinobacteriophage database (Russell and Hatfull, 2017). LadyJasley's remaining putative protein coding genes belong to phams that are highly conserved within the cluster (Table 1). Conserved phams are divided between genes coding proteins involved in structure/assembly (e.g. terminase, portal, major capsid, and minor tail), lysis (endolysin, holin), and recombination/replication (RuvC-like resolvase, DNA Polymerase I, RecB-like exonuclease/helicase, and DNA primase/helicase).

**Nucleotide sequence accession numbers**

Phage Donatella is available at GenBank with Accession No. PV876935 and Sequence Read Archive (SRA) No. SRX29291550 . Phage JellyBread is available at GenBank with Accession No. PV876964 and Sequence Read Archive (SRA) No. SRX29291525 . Phage LadyJasley is available at GenBank with Accession No. PV876980 and Sequence Read Archive (SRA) No. SRX29291530 .

Table 1. Genome characteristics of three phages infecting *Arthrobacter globiformis* B-2979.

**Table:** 

Phage	**Donatella**	**JellyBread**	**LadyJasley**
# of Reads	2,136,496	2,690,769	1,599,620
Coverage	4,230x	3,390x	3,081x
Cluster(members, to date)	FF (n = 19)	AY (n = 39)	AV (n = 7)
Genome Size, bp ^a^	42,875 (42,485 ± 692)	53,425 (52,711 ± 1,427)	45,576 (45,794 ± 583)
% GC ^a^	64.8 (65.0 ± 0.2)	62.7 (62.7 ± 0.3)	44.8 (45.6 ± 0.4)
Genome End Type	3' sticky overhang	3' sticky overhang	Direct terminal repeat
# of ORFs ^a,b^	68 (66 ± 3)	93 (96 ± 3)	60 (57 ± 1)
# of tRNAs	1	2	0
# of Highly Conserved Phams ^c^	37	36	25
# of Less Conserved Phams ^d^	30	49	1
# of Unique Phams ^e^	1	8	34

^a^ Values in parentheses indicate cluster mean and SD

^b^ Excludes duplicated genes due to direct terminal repeats

^c^ Phams present in ≥ 90% of phage genomes in cluster

^d^ Phams present in 1 – 89% of phage genomes in cluster

^e^ Phams absent in genomes of any other cluster members
